# 3,4-Methylenedioxymethamphetamine facilitates fear extinction learning

**DOI:** 10.1038/tp.2015.138

**Published:** 2015-09-15

**Authors:** M B Young, R Andero, K J Ressler, L L Howell

**Affiliations:** 1 Yerkes National Primate Research Center, Emory University, Atlanta, GA, USA; 2 Department of Psychiatry and Behavioral Science, Yerkes National Primate Research Center, Emory University, Atlanta, GA, USA; 3 Howard Hughes Medical Institute, Chevy Chase, MD, USA

## Abstract

Acutely administered 3,4-methylenedioxymethamphetamine (MDMA, ‘ecstasy') has been proposed to have long-term positive effects on post-traumatic stress disorder (PTSD) symptoms when combined with psychotherapy. No preclinical data support a mechanistic basis for these claims. Given the persistent nature of psychotherapeutic gains facilitated by MDMA, we hypothesized that MDMA improves fear extinction learning, a key process in exposure-based therapies for PTSD. In these experiments, mice were first exposed to cued fear conditioning and treated with drug vehicle or MDMA before extinction training 2 days later. MDMA was administered systemically and also directly targeted to brain structures known to contribute to extinction. In addition to behavioral measures of extinction, changes in mRNA levels of brain-derived neurotrophic factor (*Bdnf*) and *Fos* were measured after MDMA treatment and extinction. MDMA (7.8 mg kg^−1^) persistently and robustly enhanced long-term extinction when administered before extinction training. MDMA increased the expression of *Fos* in the amygdala and medial prefrontal cortex (mPFC), whereas increases in *Bdnf* expression were observed only in the amygdala after extinction training. Extinction enhancements were recapitulated when MDMA (1  μg) was infused directly into the basolateral complex of the amygdala (BLA), and enhancement was abolished when BDNF signaling was inhibited before extinction. These findings suggest that MDMA enhances fear memory extinction through a BDNF-dependent mechanism, and that MDMA may be a useful adjunct to exposure-based therapies for PTSD and other anxiety disorders characterized by altered fear learning.

## Introduction

Post-traumatic stress disorder (PTSD) is characterized by recurrent and intrusive memories for a traumatic experience. Clinical studies report long-lasting improvements in symptoms of PTSD following psychotherapy sessions paired with 3,4-methylenedioxymethamphetamine (MDMA),^1, 2^ an amphetamine derivative with mildly hallucinogenic and profound mood-elevating properties.^3^ However, no preclinical data provide a mechanistic basis for these findings. Although the most readily observable effects of MDMA are its increases in positive mood and social behavior,^3^ other mood elevators, such as anxiolytics, do not produce long-term reductions in conditioned fear or lasting positive outcomes as an adjunct to psychotherapy.^4, 5^ Given that reported improvements in PTSD symptoms following MDMA-assisted psychotherapy last for years after the intervention, we hypothesized that acute MDMA affects fear learning and molecular memory processes important in extinguishing conditioned fear responses.

Mechanisms of the learning and extinction of fear have been studied extensively in rodent models. In these models, a conditioned fear response is instated by pairing a previously neutral stimulus (conditioned stimulus; CS) and aversive stimulus (unconditioned stimulus; US), and the response is later extinguished with repeated re-exposure to the CS in the absence of the US.^6^ This is a useful model for understanding the learning and memory processes required for long-term recovery from PTSD because it resembles exposure-based therapy,^7^ wherein repeated exposure to a fear-eliciting stimulus or memory promotes a reduced fear response to future reminders of the trauma.^8^ The effect of clinically relevant doses of MDMA on either fear extinction or exposure-based therapy has not been explored to date, despite the fact that acute doses of MDMA increase serotonergic and noradrenergic signaling known to be important for fear extinction learning across species.^9, 10, 11, 12^

Recovery from PTSD and extinction learning both depend on brain-derived neurotrophic factor (BDNF). Individuals with gene variants that result in reduced release of BDNF are predisposed to developing PTSD and are less responsive to exposure-based therapy.^13, 14^ Likewise, in rodents, extinction learning is enhanced by increasing BDNF signaling in the amygdala or the medial prefrontal cortex (mPFC), regions known to be crucial for the acquisition and the long-term retention of extinction.^15, 16, 17, 18^ MDMA modulates activity in the amygdala and mPFC under resting conditions; however, its effect on brain activity during fear memory retrieval is unknown.^19^ It also remains to be explored whether acute or clinically relevant doses of MDMA alter BDNF signaling in the amygdala or mPFC on a time course that supports learning.

Here, we demonstrate in mice that MDMA improves fear extinction and acts directly upon structures and biological processes known to be crucial for extinction learning. A single dose of MDMA before fear extinction training resulted in persistent and powerful reductions in conditioned fear after extinction. Reductions in conditioned fear corresponded with rapid increases in markers of neuronal activity in the amygdala and mPFC, and increases in expression of BDNF mRNA (*Bdnf*) in the amygdala. Extinction enhancements were recapitulated when MDMA was administered directly into the basolateral subregion of the amygdala (BLA) or the infralimbic (IL) subregion of the mPFC, both of which are known to be essential for extinction learning.^20^ The effect of MDMA on extinction was completely abolished when BDNF signaling was disrupted with a neutralizing antibody in the BLA before extinction training.

The behavioral and molecular data here suggest that MDMA facilitates fear extinction-learning processes and that MDMA could serve as a useful adjunct to exposure therapy for the treatment of anxiety disorders characterized by impaired learning.

## Materials and methods

### Animals

C57BL/6 mice were from the Jackson Laboratory (Bar Harbor, ME, USA) and bred at the Yerkes National Primate Research Center at the Emory University. Mice were group-housed in ventilated cages and maintained on *ad libitum* food and water. Lights in the vivarium were turned on at 0700 hours and turned off at 2100 hours. All experiments were performed on 10- to 16-week-old male mice. Studies were performed during the light-on phase, with experiments taking place between 0900 and 1700 hours. Studies were in accordance with the National Institutes of Health guidelines, and all procedures were approved by the Institutional Animal Care and Use Committee at the Emory University.

### Drugs

*S,R*(±)-MDMA (MDMA) was supplied by the National Institute on Drug Abuse (Research Technology Branch, Research Triangle Park, NC, USA). Systemically administered MDMA was dissolved in 0.9% saline immediately before experimentation and were injected intraperitoneally at a volume of 10 μl g^−1^ body weight. Sheep anti-BDNF antibody (AB1513P) was purchased from EMD Millipore (Temecula, CA, USA). When administered centrally, MDMA was dissolved in artificial cerebrospinal fluid (119 mM NaCl, 26.2 mM NaHCO_3_, 2.5 mM KCl, 1 mM NaH_2_PO_4_, 1.3 mM MgCl_2_ and 10 mM glucose).

### Surgeries

Animals were placed under ketamine+dexmedetomidine anesthesia (100+0.5 mg kg^−1^, Zoetis, Florham Park, NJ, USA) before surgery using a stereotax. For IL infusions, a double-barreled guide cannula (C235GS system, Plastics One, Roanoke, VA, USA) was implanted. The guides were placed using the left internal guide as reference, 1.8 mm anterior to the bregma, 0.5 mm lateral and 2.5 mm ventrally. For BLA infusions, two guide cannulae mounted on a base plate (C315GS system, Plastics One) were implanted. The guides were placed 1.25 mm posterior 3.3 mm bilateral to the bregma and. The guide and dummy cannulae projected 3 mm below the base plate. After surgery, mice were given subcutaneous injections of atipamezole (5.0 mg kg^−1^, Zoetis) to reverse anesthesia and the non-steroidal anti-inflammatory meloxicam (1 mg kg^−1^) as an analgesic. Mice recovered for at least 4 days before experimentation.

### IL and BLA infusions

Habituation of mice to the investigator and the infusion procedure were carried out in two 4-min handling sessions followed by 3 min of mock infusion. Handling sessions were conducted on each of the 4 days preceding training. Mice were gently handled for 2 min 1 h before extinction training. MDMA (1 μg) or anti-BDNF antibody (0.2 μg) was infused bilaterally into IL (*n*=7 per treatment), BLA (*n*=7 per treatment) or adjacent regions (*n*=6 per site) 10 min before extinction training. All infusions were 0.2 μl per side at 0.08 μl min^−1^, and infusion cannulae were left in place for 30 s after infusion. For IL infusions, the internal guide cannula projected 0.5 mm below the base of the guide. For BLA infusions, injection cannulae extended 2.0 mm below the base of the guide. Accurate targeting was determined after the experiment by checking the placement of dye infused in the same manner as experimental drug.

### Fear conditioning and extinction

To study MDMA's effect on extinction, mice were exposed to cued fear conditioning on Day 1, fear extinction training on Day 3 and extinction testing on Day 4 (or on Day 10 to test long-term effects). Mice were habituated to handling and intraperitoneal injection for 2 days before experimentation. On habituation days, mice were handled for 4 min and injected with drug vehicle (systemic experiments) or given a simulated infusion (infusion experiments). Mice were then placed in individual plastic buckets (10.5 cm diameter at the base) with bedding and lids for 30–60 min. Before experimentation, mice were held in their respective buckets for 30–60 min. MDMA was injected intraperitoneally 30 min before training.

Cued fear conditioning consisted of a single pairing of a CS tone (75–80 dB, 4.5 kHz and 30 s) and a US footshock (1 mA and 2 s) and was carried out by placing the subject in the conditioning apparatus for 2 min before the CS tone turned on, and co-terminated with the US footshock. More extensive fear conditioning and extinction were also used for a small portion of experiments. Here, animals were conditioned to four CS–US pairings (0.6 mA and 1 s). Subjects were returned to their buckets 30 s after the last footshock, and the apparatus was cleaned with 70% EtOH between subjects. Extinction training was carried out 48 h after fear conditioning in a different context from conditioning in a different room, different training apparatus with a plexiglass floor and red light, and the apparatus was cleaned with 5% Roccal-D (Zoetis) before and after each subject. Extinction began 2 min after placing the subject in the extinction apparatus, and it consisted of a suboptimal regimen of four CS re-exposures separated by 45 s. Animals trained with more extensive conditioning were extinguished in the same manner with 14 CS tones.

Animals were returned to their home cage 30 s after the last CS exposure. Throughout these experiments, percent freezing was scored by an experimenter blind to treatment for the presence or absence of non-respiratory movement every 5 s. To test the long-term effect of extinction training on the freezing response to the CS, the exact procedure used during extinction training was repeated in the same context (or in a new context, to explore generalization of extinction) 1 or 10 days later. To test for effects on reconsolidation, mice were exposed to a single CS on Day 3 and tested on Day 4 in the same manner as previously described.

All experiments were initially performed with eight animals per group on the basis of power analyses from previous extinction experiments. Subjects were added when the initial experiment approached significance. In all experiments using fear conditioning, subjects were pseudorandomly assigned to groups so as to make total group weights equivalent to account for shock sensitivity.

### Locomotor behavior

Drug effects on locomotor activity were tested at least 10 days after the final day of fear extinction in animals that had been treated with saline. Experiments were carried out in 40 × 40 × 30-cm photocell cages (Omnitech Electronics; Columbus, OH, USA) with 32 photocells (16 front to back and 16 side to side) positioned 2.5 cm off the cage floor. Operation of the photocell cages and data collection was performed by an interfaced computer. Animals were treated with MDMA or saline (*n*=10 per treatment) 30 min before being placed in the apparatus for 10 min.

### mRNA extraction, complementary DNA synthesis and quantitative PCR

mRNA was taken from 1-mm diameter brain punches of the mPFC and amygdala. Animals were administered saline or MDMA (7.8 mg kg^−1^) 30 min before being placed in the extinction-training apparatus for ~7 min and exposed to 4 CS tones or no CS tones. Animals were killed 1 h after being removed from the extinction apparatus, and their brains were rapidly removed, flash-frozen in 2-methylbutane on dry ice and stored at −80 °C until processing. mRNA was isolated and purified from tissue punches using the RNeasy Mini Kit (catalog 74106, Qiagen). Total mRNA was reverse-transcribed with the RT^2^ First Strand Kit (catalog 330401, Qiagen). Individual samples were assessed for mRNA content using TaqMan primers for *Bdnf* Mm04230607_s1, *Fos* Mm00487425_m1 and *Gapdh* Mm03302249_g1 from Applied Biosystems. Final analyses were performed using *Gapdh* as a control. Quantitative PCR thermal cycling parameters were 10 min at 95 °C, followed by 40 cycles of amplifications for 15 s at 95 °C, 1 min at 60 °C. A dissociation stage, consisting of 15 s at 95 °C, 1 min at 60 °C and 15 s at 95 °C, was added at the end. Quantification of mRNA was performed using the Applied Biosystems 7500 Real-Time PCR System. Samples were excluded if control measures (*Gapdh*) were not within the expected range, or if experimental measures were more than two s.d.'s from the mean.

### Statistical analysis

Behavioral data were analyzed with SPSS 22.0 (IBM, Armonk, NY, USA) and Prism 5.0 (GraphPad, La Jolla, CA, USA) using a one-tailed *t*-test, one- or two-way analysis of variance (ANOVA) or a repeated-measures ANOVA with *α*=0.05. *Post hoc* ANOVA comparisons were made using Dunnett's test for dose–response data and Bonferroni's test for all other data. Data for quantitative PCR were compared against saline-treated controls and analyzed with a *t*-test. Data in figures are presented as mean±s.e. The data variance was similar between the groups compared in each statistical analysis using Bartlett's test for equal variances.

## Results

### MDMA persistently enhances fear extinction when given before extinction training

**Figure 1:**
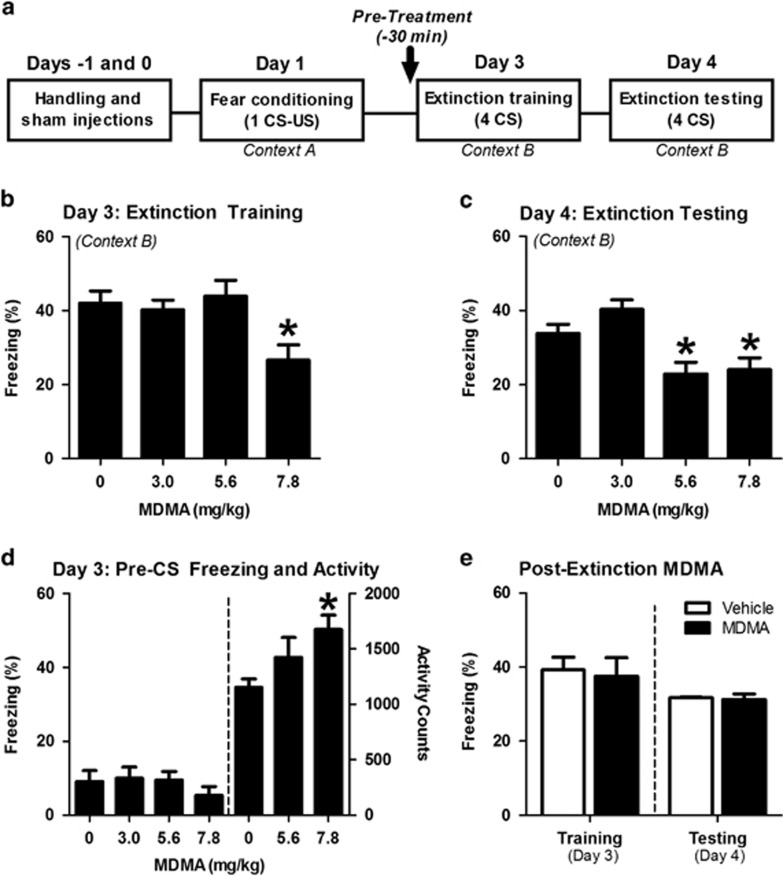
3,4-Methylenedioxymethamphetamine (MDMA) treatment before extinction training reduced conditioned freezing 24 h later. Unless otherwise noted, all treatments were given intraperitoneally 30 min before extinction training on Day 3. (**a**) Primary behavioral procedure for exploring MDMA's effect on fear extinction. (**b**) MDMA dose-dependently decreased freezing to the tone during extinction training across four CS tones (F(3,30)=4.58, *P*=0.0093 for main effect of dose; *n*=8–10 per group). (**c**) Animals previously extinguished under MDMA exhibited dose-dependent reductions in freezing to four CS tones 24 h later (F(3,30)=8.30, *P*=0.0004 for main effect of dose; *n*=8–10 per group). (**d**) MDMA did not affect freezing before the first CS-tone re-exposure (F(3,30)=0.67, *P*=0.5742 for main effect of treatment; *n*=8–10/group), but 7.8 mg kg^−1^ MDMA increased locomotor activity in a 10- min test (F(2,26)=3.62, *P*=0.0411 for main effect of dose; *n*=9–10 per group). (**e**) MDMA (7.8 mg kg^−1^) administered immediately following extinction training on Day 3 did not affect freezing in response to four CS tones the following day (*t*(10)=1.19, *P*=0.26 for effect of treatment; *n*=6 per group). Bars represent mean±s.e. values of %freezing. *P<0.05. CS, conditioned stimulus; US, unconditioned stimulus.

The effect of MDMA on fear extinction was investigated using a version of the Pavlovian-cued fear conditioning and extinction paradigm (Figure 1a). MDMA given 30 min before extinction training reduced freezing to the CS tone during the session, with 7.8 mg kg^−1^ significantly reducing freezing (Figure 1b). Twenty-four hours later, tested in the extinction context in a drug-free state, mice previously given either 5.6 or 7.8  mg kg^−1^ MDMA before extinction training exhibited similar reductions of conditioned freezing (Figure 1c). MDMA (7.8 mg kg^−1^) increased locomotor behavior but did not alter freezing in the extinction apparatus during the 2 min before the first CS-tone exposure (Figure 1d). No reductions in conditioned freezing were observed when MDMA was administered immediately after extinction training (Figure 1e).

**Figure 2:**
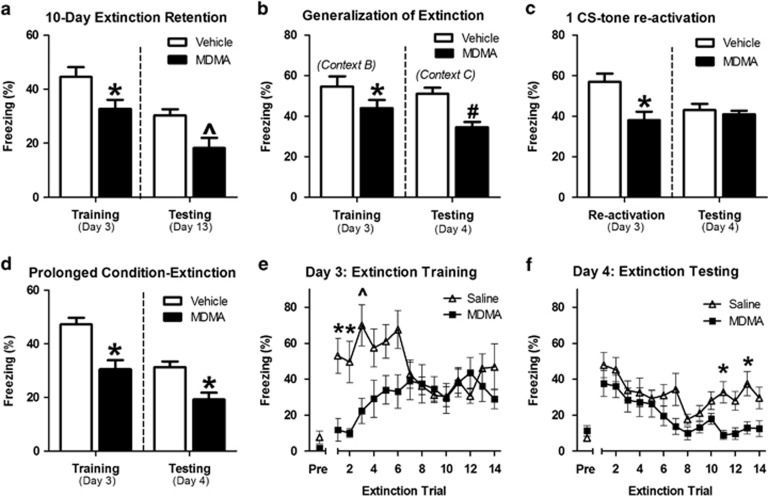
3,4-Methylenedioxymethamphetamine (MDMA) pre-treatment has prolonged and generalized effects on extinction. MDMA (7.8 mg kg^−1^) was administered 30 min before re-exposure to the CS tone on Day 3. (**a**) MDMA-treated mice exhibited a greater reduction in freezing to four CS tones 10 days later (*t*(14)=3.05, *P*=0.0093 for effect of treatment; *n*=8 per group). (**b**) MDMA-treated animals exhibited reduced freezing to four CS tones when tested outside of the extinction context 24 h later. (*t*(14)=4.45, *P*=0.0006 for effect of treatment; *n*=8 per group). (**c**) The effect of MDMA pre-treatment on the response to a single CS-tone re-exposure was tested. MDMA reduced freezing during re-activation (*t*(8)=2.77, *P*=0.0122 for effect of treatment; *n*=5 per group) but did not affect conditioned freezing the following day (*t*(8)=0.66, *P*=0.2633 for effect of treatment; *n*=5 per group). (**d**) Animals were conditioned to four CS–US pairings (0.6 mA, 0.5 s). MDMA administered before extinction training with 14 CS tones significantly reduced conditioned freezing during the extinction session (*t*(16)=2.21, *P*=0.0280 for effect of MDMA; *n*=8–10 per group) and 24 h later when animals were tested with 14 CS tones (*t*(16)=2.679, *P*=0.0150 for effect of MDMA; *n*=8–10 per group). (**e**) MDMA-treated animals exhibited significantly less freezing during the first six CS exposures of prolonged extinction training (F(1,16)=5.80, *P*=0.0280 for between-subject effect of treatment). (**f**) Pre-extinction MDMA reduced conditioned freezing the following day (F(1,16)=7.24, *P*=0.0150 for between-subject effect of treatment). Bars represent mean±s.e. values of %freezing. *P<0.05, ^*P*<0.01, ^#^*P*<0.001. CS, conditioned stimulus; US, unconditioned stimulus.

To determine the durability of MDMA's effect, we tested whether it persisted in response to manipulations known to interfere with fear extinction. Many drugs fail to maintain extinction enhancements for extended periods of time. Here, extinction enhancements observed in MDMA-treated animals 24 h after extinction training persisted for at least 10 days (Figure 2a). Following fear extinction, the formerly conditioned fear response can be renewed if the subject is re-exposed to the CS anywhere but the extinction context.^21^ Here, extinction enhancements induced by MDMA persisted when extinguished animals were tested in an unfamiliar context (Figure 2b).

Given that the extinction-training paradigm used here is shorter than those used in some other studies, we explored whether MDMA's effect on conditioned freezing were because of impairments of reconsolidation. Administering MDMA 30 min before a single CS-tone re-exposure on Day 3 decreased conditioned freezing during the initial trial but did not affect conditioned freezing the following day (Figure 2c), suggesting that MDMA did not impair reconsolidation. We further demonstrated MDMA's effects on extinction by using a more extensive fear-conditioning (four CS–US pairings; 0.6 mA, 1 s) and suboptimal extinction paradigm.^22^ Animals treated with MDMA 30 min before re-exposure to 14 CS tones exhibited significantly less freezing during the first six CS tones; however, their behavior returned to control levels by the end of the trial. The following day, MDMA-treated animals exhibited overall decreases in conditioned freezing to 14 CS tones (Figure 2d), and in particular to the final re-exposures.

### MDMA treatment that enhances extinction increases neurobiological markers of extinction learning

**Figure 3:**
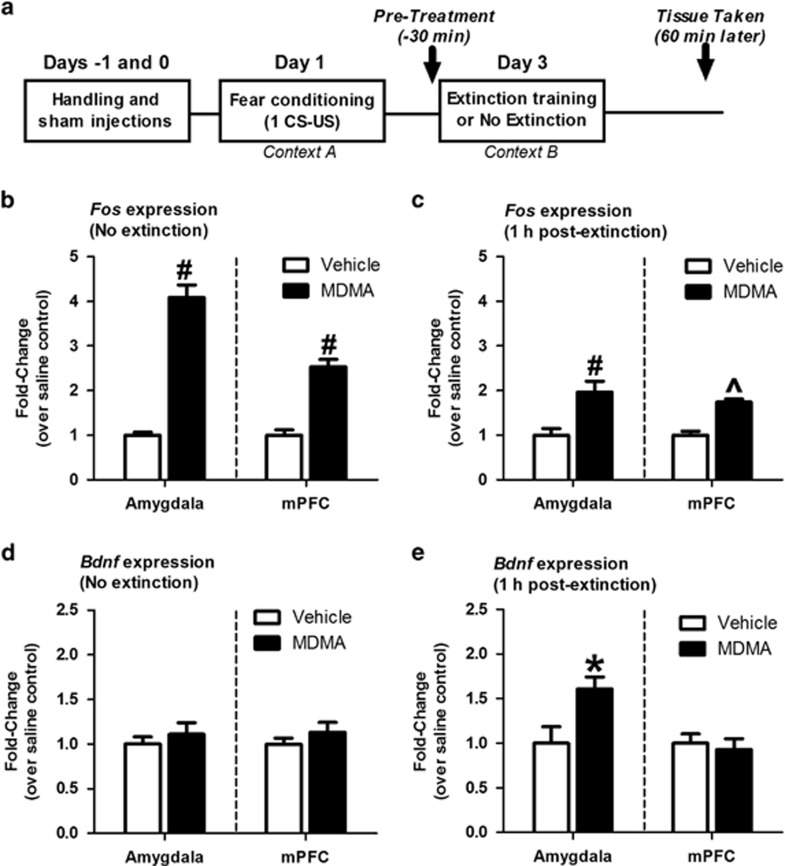
3,4-Methylenedioxymethamphetamine (MDMA) increases *Bdnf* and *Fos* mRNA expression in the amygdala and medial prefrontal cortex (mPFC). (**a**) Behavioral and experimental procedures for assessing the effect of MDMA and extinction on mRNA levels. (**b**) MDMA pre-treatment increased *Fos* mRNA in the amygdala and mPFC 1 h after placing animals in the extinction apparatus without extinction training (*t*(18)=11.29, *P*<0.0001 for effect of MDMA in amygdala; *t*(18)=6.915, *P*<0.0001 for effect of MDMA in mPFC; *n*=9–10 per group). (**c**) *Fos* expression was increased in the amygdala and mPFC after combining MDMA with extinction training (*t*(28) 3.428, *P*=0.0019 for effect of MDMA in the amygdala; *t*(17)=6.258, *P*<0.001 for effect of MDMA in mPFC; *n*=9–16 per group). (**d**) MDMA (7.8 mg kg^−1^) pre-treatment did not alter *Bdnf* expression in the amygdala or mPFC but (**e**) increased *Bdnf* mRNA in the amygdala 1 h after extinction training (*t*(28)=2.469, *P*=0.0199 for effect of MDMA; *n*=16 per group). Bars represent mean±s.e. values of mRNA levels. *P<0.05, ^*P*<0.01, ^#^*P*<0.001. *Bdnf*, brain-derived neurotrophic factor; CS, conditioned stimulus; mPFC, medial prefrontal cortex; US, unconditioned stimulus.

To explore whether long-lasting MDMA-induced reductions in conditioned freezing correspond with molecular learning processes, we measured changes in expression in the early-response gene *Fos* in response to MDMA and/or extinction training. MDMA increased levels of *Fos* mRNA in both the mPFC and amygdala ~97 min after administration, regardless of whether animals were exposed to extinction training (Figures 3b and c).

We also explored whether MDMA treatments that enhance extinction also increase BDNF signaling. Animals were treated with MDMA or saline (−30 min), and levels of BDNF mRNA (*Bdnf)* were measured in the amygdala or mPFC 1 h after extinction training. Control animals were exposed to the extinction apparatus without CS presentation. A dose of MDMA sufficient to enhance extinction had no effect on *Bdnf* mRNA levels in either the mPFC or amygdala of non-extinguished control animals (Figures 3d and e). However, *Bdnf* expression was increased in the amygdala when MDMA was combined with extinction training (Figure 3e). No differences in *Bdnf* expression were observed in the mPFC of saline- and MDMA-treated animals after extinction training (Figure 3d). Therefore, combining MDMA with extinction training increases *Bdnf* expression in the amygdala and facilitates fear extinction.

### MDMA enhances fear extinction via the IL cortex and basolateral amygdala

The amygdala and mPFC consist of a number of subregions with unique behavioral functions. Given that samples taken from the amygdala and mPFC for quantitative PCR encompassed the whole of each structure, we sought to determine whether MDMA exerts its behavioral effects in the IL cortex of the mPFC and the BLA, subregions required for extinction learning.^23^

**Figure 4:**
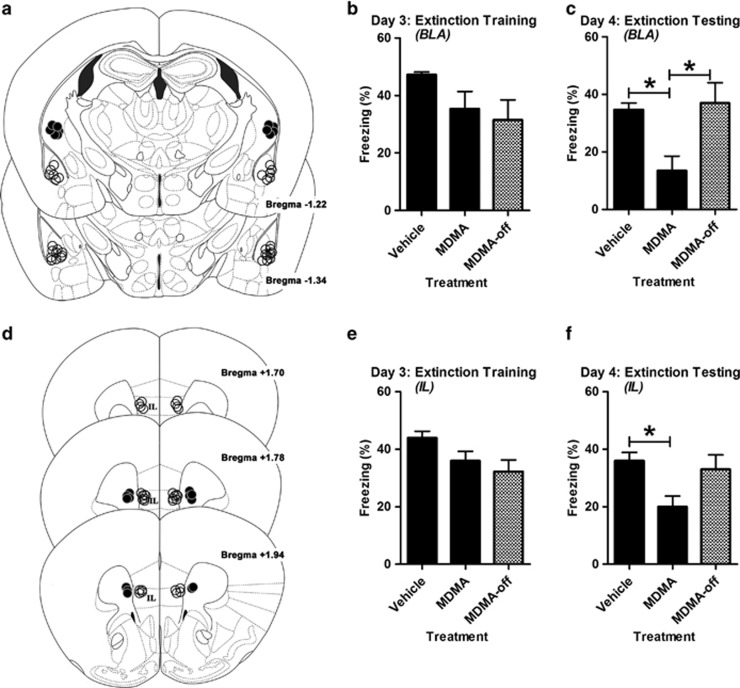
Infusing 3,4-methylenedioxymethamphetamine (MDMA) into the basolateral amygdala (BLA) or infralimbic (IL) cortex enhances extinction training. MDMA (1 μg) was infused bilaterally 10 min before extinction training on Day 3. (**a**) Infusion locations in the BLA. Open circles (○) represent on-target infusions. Black circles (**●**) indicate off-target infusions aimed 0.75 mm dorsal to the BLA. (**b**) Infusing MDMA into the BLA did not significantly reduce conditioned freezing to the CS tone during extinction training (F(2,19)=2.59, *P*=0.102 for effect of treatment; *n*=6–8 per group). (**c**) Infusing MDMA into the BLA before extinction significantly reduced conditioned freezing 24 h later (F(2,19)=7.47, *P*=0.004 for effect of treatment; *n*=6–8 per group). (**d**) Infusion locations in the IL. Black circles (**●**) indicate off-target infusion aimed 0.5 mm medial of the IL. (**e**) Infusing MDMA into IL did not significantly reduce conditioned freezing to the CS tone during extinction training (F(2,17)=3.50, *P=*0.0534 for effect of treatment; *n*=6–7 per group). (**f**) Infusing MDMA into the IL before extinction significantly reduced conditioned freezing 24 h later (F(2,17)=4.89, *P*=0.021 for effect of treatment; *n*=6–7 per group). Bars represent mean±s.e. values of %freezing. *P<0.05. CS, conditioned stimulus.

Bilateral infusions of MDMA (1 μg per side) into the IL or BLA 10 min before extinction training did not reduce conditioned freezing to the CS tone during the extinction training session compared with vehicle-treated controls or animals given MDMA in regions adjacent to the target region (Figures 4b and e). Both BLA and IL treatments significantly reduced freezing 24 h later compared with vehicle-treated controls or animals treated with off-target MDMA (Figures 4c and f).

### Extinction enhancements by MDMA in the BLA require intact BDNF signaling

**Figure 5:**
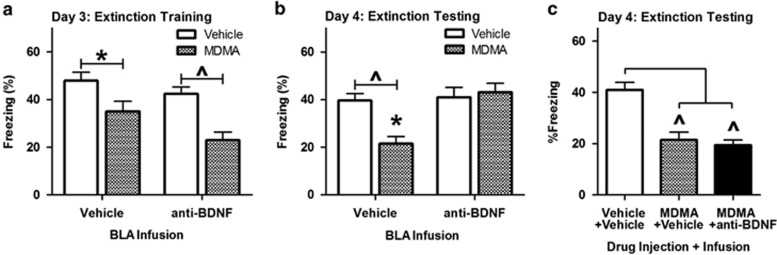
Interrupting brain-derived neurotrophic factor (BDNF) signaling in the basolateral complex of the amygdala (BLA) blocks 3,4-methylenedioxymethamphetamine's (MDMA's) extinction-enhancing effect. MDMA (7.8 mg kg^−1^) was administered systemically 30 min before extinction training. Anti-BDNF (0.2 μg) was infused bilaterally into the BLA 10 min before extinction training. (**a**) Anti-BDNF did not alter MDMA's effect on conditioned freezing during extinction training (F(1,20)=27.40, *P*<0.001 for the main effect of systemic treatment; (F(1,20)=0.458, *P*=0.506 for effect of infusion treatment; *n*=6 per group). (**b**) Anti-BDNF treatment before extinction training blocked MDMA-induced reductions in conditioned freezing during testing the following day (F(1,20)=4.62, *P* =0.044 for main effect of injection treatment; F(1,20)=9.58, *P*=0.006 for main effect of infusion; F(1,20)=7.38, *P*=0.013 for interaction of injection and infusion treatment; *n*=6 per group). (**c**) Anti-BDNF did not block MDMA's effect when it was given 10 h after extinction training (F(2,15)=11.86, *P*=0.01 for effect of treatment; *n*=6 per group). Bars represent mean±s.e. values of %freezing. *P<0.05, ^*P*<0.01, ^#^*P*<0.001.

To determine whether extinction enhancements mediated by MDMA in the BLA depend on BDNF there, we obstructed BDNF signaling before extinction by directly infusing a BDNF-neutralizing antibody. Following systemic MDMA treatment, anti-BDNF (0.2 μg per BLA) was administered 10 min before extinction training. This dose of anti-BDNF was used on the basis of studies in which the same concentration of the antibody inhibited BDNF and aversive learning.^20, 24^ Anti-BDNF treatment did not alter the reductions in freezing induced by 7.8 mg kg^−1^ of MDMA during extinction training (Figure 5a). The following day, reductions in conditioned freezing elicited by MDMA treatment were abolished in animals that had been treated with anti-BDNF before extinction training (Figure 5b). Anti-BDNF treatment did not alter conditioned freezing in animals that did not receive MDMA, indicating that anti-BDNF itself did not affect conditioned freezing following suboptimal extinction. To rule out possible effects of lingering bioavailable anti-BDNF during extinction testing, anti-BDNF was administered to MDMA-treated animals 10 h after extinction training. Anti-BDNF infusion 10 h after extinction did not interrupt the effect of MDMA on conditioned freezing during extinction testing (Figure 5c).

## Discussion

Few, if any, pharmacological treatments alleviate the recurrent and intrusive traumatic memories characteristic of PTSD. Given the long-lasting alleviation of PTSD symptoms following MDMA-assisted psychotherapy, we asked whether MDMA has an effect on extinction learning.^2^ We demonstrate here that MDMA facilitates fear extinction and neurobiological processes associated with extinction learning. Specifically, MDMA treatment before extinction training induced long-term reductions in conditioned fear that persisted even when the fear-eliciting stimulus was presented in a novel context. Directly infusing MDMA into the IL or BLA, but not into adjacent regions, also enhanced extinction. Extinction enhancement by MDMA coincided with increased markers of neuronal activity in the amygdala and mPFC, as well as increased expression of *Bdnf* in the amygdala. Disrupting BDNF signaling in the BLA completely abolished MDMA's effect on extinction. Together, these data demonstrate that MDMA enhances fear extinction learning in a BDNF-dependent manner.

MDMA facilitated the extinction of conditioned fear whether animals were initially conditioned with either a single CS–US pairing or multiple CS–US pairings. Several manipulations fail to conserve their effects on fear learning when tested across different fear-conditioning protocols, as single and multiple CS–US training paradigms appear to engage unique profiles of neurobiological mechanisms.^25, 26^ Here, we demonstrate that MDMA reduced conditioned freezing during extinction testing overall. In the multiple CS–US-trained animals, conditioned freezing was most significantly reduced during the final five CS tones of extinction testing, suggesting that MDMA increases the capacity of extinction. Interestingly, reductions in freezing during extinction training did not appear to be necessary for MDMA's long-term effects on extinction. Whereas MDMA robustly reduced conditioned freezing during the first half of extinction training of multiple CS–US pairings, we believe these reductions to be attributable to MDMA's psychostimulant properties.^9^ Central infusion of MDMA or a systemically administered dose of 5.6 mg kg^−1^ both reduced freezing during extinction testing without affecting freezing during extinction training. In addition, the same dose of MDMA that reduced conditioned freezing during extinction training also increased locomotor behavior across the same period of time. Our hypothesis that reductions in freezing are due to MDMA's psychostimulant properties, and not to an effect on extinction acquisition, is consistent with previous research demonstrating that stimulants can robustly reduce conditioned freezing during extinction training without maintaining that effect during later testing.^27^

Reductions in conditioned freezing were not only observed the day after treatment and extinction, but for several days afterward. Most strikingly, reductions in conditioned freezing also persisted when the CS tone was presented in an unfamiliar context. Typically, re-exposure to the CS tone in a new context renews the fear response to the CS.^21^ Ablation of fear renewal in a novel context typically requires massive extinction protocols or extinction training in multiple contexts.^28, 29^ That a similar context generalization was observed following a less intensive extinction paradigm demonstrates that MDMA induces a powerful form of extinction learning potentially translatable to more complex circumstances in human PTSD patients.

Our results inform clinical studies by suggesting that MDMA must be on-board during extinction training to induce extinction enhancements. This appears to be due to an effect between MDMA and activity-dependent processes in the amygdala that drive subsequent BDNF signaling important for consolidation. BDNF signaling is a crucial molecular signature of several forms of learning.^30^ Treatments that increase BDNF signaling enhance extinction learning,^17, 31^ whereas treatments that impair BDNF signaling impair it.^32, 33^ We observed that MDMA administration increases *Bdnf* expression in the amygdala only when combined with extinction training. The effect of combined MDMA and extinction training on *Bdnf* expression may explain why post-training MDMA administration did not induce the same extinction enhancements as pre-training administration. Extinction-dependent increases in *Bdnf* mRNA induced by MDMA suggest that its effect on extinction is a learning phenomenon. This is consistent with reports that BDNF is required in the BLA for consolidation of extinction memory,^32^ and that chronically administered selective-serotonin reuptake inhibitors reduce conditioned fear only when combined with extinction training.^34^

Acutely administered MDMA (10 mg kg^−1^) has been previously observed to increase *Bdnf* expression in the mPFC 24 h later.^35^ Although we did not observe such changes here, BDNF in the IL is known to facilitate fear conditioning.^18^ Therefore, other unobserved MDMA-induced changes in BDNF signaling that also contribute to extinction enhancement may occur at a later time. Many extinction-induced increases in BDNF are observed after 2 h in a variety of brain regions.^31, 36^ However, our observation that disrupting BDNF signaling in the BLA abolished the effect of systemically administered MDMA underscores the importance of BDNF in the BLA.

Given the wide variety of MDMA's neuropharmacological effects, it is difficult to speculate on how it might rapidly increase extinction learning and *Bdnf* expression. MDMA's primary effect is to release serotonin (5-HT) and norepinephrine (NE),^9^ which is essential to MDMA's acute behavioral effects.^37^ Increasing 5-HT and NE with reuptake inhibitors upregulates *Bdnf* expression, but only with chronic regimens.^38^ Similarly, only chronic regimens of 5-HT reuptake inhibitors enhance fear memory extinction.^34^ MDMA rapidly and powerfully increases extracellular monoamine levels by reverse transport,^39^ and therefore acute treatment may achieve increases in 5-HT and NE that typically require chronic administration of transporter inhibitors. MDMA also binds directly to 5-HT_2A_ receptors that have been observed to enhance fear memory extinction^40, 41^ and increase BDNF signaling.^42^ Given MDMA's combined abuse liability and neurotoxicity,^43^ it is important to isolate the pharmacological mechanisms through which MDMA enhances extinction in order to develop drugs with fewer risks.

For a brief period beginning in the 1970s, MDMA was being explored as an adjunct to psychotherapy, primarily for inducing mildly altered states of consciousness with increased social/empathic behaviors and reduced anxiety.^3, 44, 45^ Whereas the general pharmacology of MDMA's effects on subjective states have been explored, these have provided little information on how acute MDMA experiences induce long-lasting behavioral changes, such as those observed in PTSD patients treated with MDMA.^2^ Our observations suggest that MDMA enhances fear extinction learning in part by upregulating BDNF signaling in the amygdala. Further clinical studies of MDMA as an adjunct to exposure therapy should be explored, as impairments in activity-dependent BDNF release contribute to extinction-learning deficits in PTSD.^13, 14^
